# Heterotopic Mesenteric Ossification With Trilineage Hematopoiesis

**DOI:** 10.7759/cureus.24620

**Published:** 2022-04-30

**Authors:** Emily M Martinbianco, Cullen M Lilley, Joseph Grech, Kamran M Mirza, Xiuxu Chen

**Affiliations:** 1 Department of Pathology, Loyola University Chicago Stritch School of Medicine, Maywood, USA; 2 Department of Pathology and Laboratory Medicine, Loyola University Medical Center, Maywood, USA

**Keywords:** extramedullary hematopoiesis, metaplastic ossification, myositis ossificans, ectopic bone formation, mesenteric ossification, heterotopic ossification

## Abstract

Heterotopic ossification (HO) histologically refers to extraskeletal bone formation in non-ossifying tissues, most commonly noted in the extremities, buttocks, abdominal wall, and hip joints. HO developing in the mesentery (heterotopic mesenteric ossification, HMO) is very rare, with fewer than 100 cases reported in the literature. It usually occurs in adult male patients with a history of repeated abdominal trauma. So far, only two cases of HMO have been reported with the development of hematopoietic bone marrow. Here, we report the third case of HMO with true trilineage hematopoiesis in a 66-year-old female with suspicious mesenteric-retained foreign material from prior surgical procedures, including hysterectomy for endometrial adenocarcinoma and multiple repairs for incisional hernia.

## Introduction

Heterotopic ossification (HO) refers histologically to extraskeletal bone formation in non-ossifying tissues with a yet to be determined incidence, exact etiology, and pathogenesis [1-4]. It was initially reported in 1894 in the inner thigh of horseback riders, presumably because of saddle trauma [5]. There are two primary forms of HO, namely, genetic and acquired. The former refers to rare hereditary diseases such as myositis ossificans progressiva (MOP, also known as fibrodysplasia ossificans progressiva), Albright’s hereditary osteodystrophy, and progressive osseous heteroplasia [4]. The latter includes a heterogeneous group of conditions of ectopic ossification related to traumatic or neurogenic etiologies, such as extrinsic trauma, burn, surgeries, spinal cord or brain injuries, and others [3,6,7]. Although HO may occur at various sites of the body, it is most commonly seen in the extremities [8]. HO involving the mesentery, termed heterotopic mesenteric ossification (HMO) by Wilson et al. [5] (also referred to as intra-abdominal myositis ossificans, mesenteritis ossificans, heterotopic ossificans of the intestinal mesentery [9]), is even rarer, with fewer than 100 cases published since the first report by Hansen et al. in 1983 [8,10-12]. Of all HO cases, only nine cases reported the presence of bone marrow components, including two cases with adipose tissue [12,13] and seven cases of true hematopoiesis (six cases of non-mesenteric HO [7,14-18] and two cases of HMO [10,19]). Here, we report the third case of HMO with true trilineage hematopoiesis in a 66-year-old female following multiple abdominal surgeries.

## Case presentation

**Figure 1:**
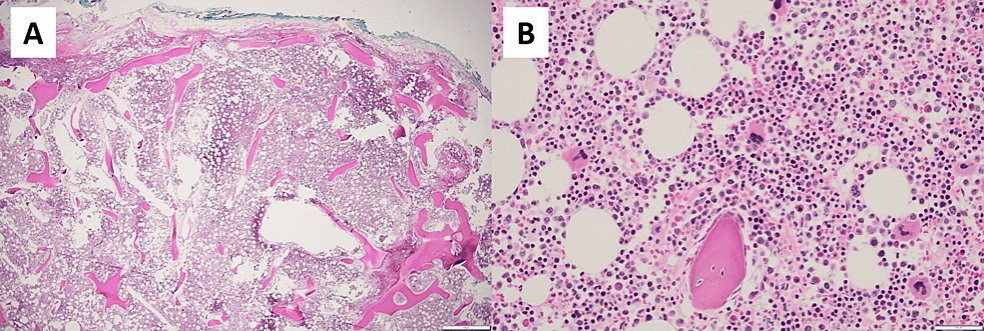
Photomicrographs for HMO with true trilineage hematopoiesis. A: The 1.5-cm well-circumscribed firm mass in the mesentery was confirmed to be an HMO with bone trabeculae and well-developed bone marrow at low-power magnification. B: The high-power field demonstrates that the trabeculae are composed of mature cortical lamellar bone with focally rimming osteoblasts, mature osteocytes in lacunae and cement line, and true trilineage hematopoiesis with erythropoiesis, granulocytopoiesis, and megakaryocytopoiesis among adipocytes. HMO: heterotopic mesenteric ossification

This case is of a 66-year-old female with a clinical history of hypertension, diabetes, obesity, and endometrial adenocarcinoma status post-total hysterectomy 10 years ago. Because of the surgery, she developed an abdominal incisional hernia that was treated with multiple repair surgeries, complicated by intra-abdominal infection, septic shock, and acute renal and respiratory failure. She subsequently presented with recurrent incisional hernia with strangulated small intestine and obstruction and was treated with right hemicolectomy and ileostomy. Differential blood count one week before revealed severe anemia (white blood cell count, 6,000/µL; red blood cell count, 2.88 × 10^6^/µL; hemoglobin, 7.6 g/dL; hematocrit, 23.5%; mean corpuscular volume, 81.6 fL; mean corpuscular hemoglobin, 26.3 pg; mean corpuscular hemoglobin concentration, 32.2 g/dL; red cell distribution width, 15.4%; platelet count, 145 k/µL, mean platelet volume, 8.2 fL). Computed tomography of the abdomen reported a linear opacity adjacent to the gastric body and antrum, as well as a high-density mass in the mesentery suspicious for possibly retained foreign body. After five abdominal surgeries for a complicated postoperative course, she fully healed and underwent an ileostomy take-down one month later. During the surgery of ileostomy take-down, the mesentery appeared markedly congested and slightly adhered to the anterior abdominal wall. An intra-abdominal mass was palpated in the mesentery. Pathological gross examination showed that the excised mass was well-circumscribed and firm, located in fibrous and adipose tissue, and measured 1.5 cm in the greatest dimension with a whitish trabeculated cut surface. Microscopic examination showed mature cortical lamellar bone with osteocytes, cement line and rimming osteoblasts, and true trilineage hematopoiesis with erythropoiesis, granulocytopoiesis, and megakaryocytopoiesis (Figure 1). The diagnosis of a retained foreign body was excluded and a definitive diagnosis of HMO was rendered. The patient healed well from ileostomy reversal with an ensuing large ventral hernia to repair.

## Discussion

HMO is a rare subtype of HO occurring in the mesentery. The exact incidence and prevalence rates for HMO are unclear because many cases are believed to be asymptomatic and underdiagnosed [16]. It is believed to be relatively rare because only fewer than 100 cases have been reported so far in the English-language literature [12]. An analysis of pooled cases from 1983 [8,10] to 2022 showed a median age of 49.5 years, predominantly male (92.9% or 65/70), common history of previous surgery or trauma (93.0% or 66/71), a median interval of approximately four weeks between last procedure/trauma to the diagnosis of HMO, reported marrow component (16.7% or 3/18), and most common complication of small bowel obstruction (45.3% or 29/64) [5,8,10,12,13,19-26].

The most frequently reported radiological features of HMO, similar to other sites in HO, are dense curvilinear trabecular opacities with well-defined cortex [13,22] or mesh-like lesions in some cases [24]. Histologically, the typical feature of zonation was recognized in 1958 by Ackerman who described three distinct zones in HO, a central cellular proliferation zone, middle oriented osteoid formation zone with osteoblastic rimming, and a peripheral zone with mature lamellar bone [1]. This zonation phenomenon implies a benign process of progressive orientation and maturation of bone formation in HO and was confirmed by subsequent reports [5,8].

Two hypotheses have been proposed to explain the possible pathogenesis of HO. Based on the finding that HO was exclusively identified in vertical but not horizontal, abdominal wall incision scars in 23 cases, Marteinsson and Musgrove proposed that accidental seeding of osteogenic cells from periosteum or perichondrium of xiphoid process or symphysis pubis was the most likely mechanism for the development of HO [6]. However, this hypothesis does not explain the occurrence of HO in certain cases. For example, HO was identified in the inferior vena cava, where no anatomic osseous tissue was involved [18], or in cases that had no previous surgical or trauma history [12]. The low incidence rate of HO in orthopedic patients also argues against this hypothesis, considering the number of orthopedic procedures performed in clinical practice. The second hypothesis is the differentiation of multipotent mesenchymal stem cells into osteogenic cells under certain conditions [6,13]. Kaplan et al. and others developed the theory further by proposing that four factors were required for HO to develop, including a persistent inciting event such as repeated trauma or multiple surgeries, activating signal(s), multipotent mesenchymal stem cells, and a permissive microenvironment [14,26-28]. Hypoxia, hypercalcemia, change of sympathetic nerve activity, and mobilization/immobilization may also contribute to heterotopic ossification [14]. Interestingly, a potential role for the bone morphogenic protein (BMP) signaling pathway, including multiple BMPs and their negative antagonists (noggin, gremlin, follistatin, and chordin), has been suggested in the development of HO by several studies [5,12,14,24], especially BMP-4 and BMP-1 receptor ACVR1 gene mutation in MOP [29]. These hypotheses are plausible, key molecule(s) to determine this peculiar pathway from tissue injury to ectopic osseous formation, however, is still a mystery. More studies are needed to elucidate the mechanism of the heterotopic ossification process.

Production of bone marrow with trilineage hematopoiesis in HMO is even rarer. Only three HMO cases including our case reported marrow components in the literature [10,19]. Similar to the pathogenesis of HO, two hypotheses for the development of true trilineage hematopoiesis in HMO have been proposed: implantation of circulating peripheral blood CD34+ hematopoietic stem cells (“seeding pathway”), or sharing on-site progenitor cells between ossification and hematopoiesis that differentiate into both osteogenic cells and hematopoietic cells (“on-site pathway”). The currently well-established bone marrow transplantation using CD34+ stem cells in clinical practice and the potential challenge of microenvironmental control in the “on-site pathway” conceptually favor the former. However, either pathway cannot be excluded until evidence emerges in the future.

The main clinical significance for recognizing HMO is its complications, such as small bowel obstruction, ileus, abortion of surgical procedure due to extensive involvement of mesenteric vasculature [5,8,17,25], and its differential diagnosis from malignancies such as peritoneal carcinomatosis which is important due to relevance in clinical management. Other differential diagnoses include dystrophic calcification (no osteoblasts), leakage of contrast, and foreign materials [12,13,17,22,24]. Clinical management of HMO depends on the presence of symptoms and complications. The surgical procedure to remove the HMO might be necessary for symptomatic patients or for those with severe complications such as ileus or obstruction. Invasive treatment is considered unnecessary for asymptomatic patients [17]. Noninvasive treatments, such as non-steroidal anti-inflammatory drugs (e.g., ibuprofen, indomethacin), diphosphonates (etidronate), or radiation therapy have been reported in early studies [2,7,10], but are rarely seen in recent literature [13,30].

## Conclusions

HMO refers to a rare subtype of HO in the mesentery with an unknown etiology. It is predominantly seen in adult male patients with repeated abdominal trauma. Severe complications such as ischemia and small bowel obstruction may occur in HMO patients. Radiological and histological findings of typical zonation phenomenon are helpful to differentiate HMO from other mimics such as malignancy. HMO with true trilineage hematopoiesis is extremely rare. Although multiple hypotheses have been proposed to explain the formation of HMO, underlying molecular pathway(s) for both HMO and ectopic marrow development remain to be elucidated.
